# Enemas with mesalazine increase the tissue contents of mucins in the colonic mucosa devoid of fecal stream^1^

**DOI:** 10.1590/s0102-865020190040000006

**Published:** 2019-04-29

**Authors:** Carlos Augusto Real Martinez, Fábio Guilherme Campos, Danilo Toshio Kanno, Eli Cristiano Meneses, Gabrielle Maira Matijascic, Eduardo Felipe Kim Goto, José Aires Pereira

**Affiliations:** I PhD, Associate Professor, Postgraduate Program in Health Sciences, Universidade São Francisco (USF), Bragança Paulista-SP, and Department of Surgery, Universidade Estadual de Campinas (UNICAMP), Campinas-SP, Brazil. Conception and design of the study, statistics analysis, interpretation of data, manuscript preparation and writing, critical revision.; II PhD, Associate Professor, Department of Gastroenterology, Faculty of Medicine, Universidade de São Paulo (USP), Brazil. Interpretation of data, critical revision.; III Fellow Master degree, Assistant Professor, Division of Surgery, Faculty of Medicine, USF, Bragança Paulista-SP, Brazil. Technical procedures, acquisition of data.; IV Fellow Master degree, Assistant Professor, Faculty of Pharmacology, USF, Bragança Paulista-SP, Brazil. Technical procedures, acquisition of data.; V Graduate student, Faculty of Medicine, USF, Bragança Paulista-SP, Brazil. Technical procedures, acquisition of data.; VI PhD, Assistant Professor, Division of Pathology, Faculty of Medicine, USF, Bragança Paulista-SP, Brazil. Histopathological examinations, acquisition and interpretation of data.

**Keywords:** Mesalamine, Colitis, Fatty Acids, Volatile, Mucins, Image Processing, Computer-Assisted, Rats

## Abstract

**Purpose:**

To evaluate the inflammatory reaction and measure the content of mucins, in the colonic mucosa without fecal stream submit to intervention with mesalazine.

**Methods:**

Twenty-four rats were submitted to a left colostomy and a distal mucous fistula and divided into two groups according to euthanasia to be performed two or four weeks. Each group was divided into two subgroups according daily application of enemas containing saline or mesalazine at 1.0 g/kg/day. Colitis was diagnosed by histological analysis and the inflammatory reaction by validated score. Acidic mucins and neutral mucins were determined with the alcian-blue and periodic acid of Schiff techniques, respectively. Sulfomucin and sialomucin were identified by high iron diamine-alcian blue technique. The tissue contents of mucins were quantified by computer-assisted image analysis. Mann-Whitney test was used to analyze the results establishing the level of significance of 5%.

**Results:**

Enemas with mesalazine in colonic segments without fecal stream decreased the inflammation score and increased the tissue content of all subtypes of mucins. The increase of tissue content of neutral, acid and sulfomucin was related to the time of intervention.

**Conclusion:**

Mesalazine enemas reduce the inflammatory process and preserve the content of mucins in colonic mucosa devoid of fecal stream.

## Introduction

 There is increasing evidence suggesting that regular production of short-chain fatty acids (SCFAs) as critical role to preserve the integrity of the colonic epithelial barrier^1^. Acetate (C2), propionate (C3) and, particularly butyrate (C4) are the main types of SCFAs produced during the fermentation of indigestible saccharides by specific colonic anaerobic bacteria’s present colonic microbiota^2^. Butyrate is the major and preferred metabolic substrate of the colonic epithelial cells providing at least 60-70% of their energy requirements necessary for their proliferation and differentiation^3^. Butyrate induces trophic effects on colonic epithelium and exerts potent effects on a variety of colonic mucosal functions such as inhibition of inflammation and carcinogenesis, reinforcing various components of the colonic defense barrier^4^. SCFAs acts as mediators of several immune processes, and reduces the production of inflammatory cytokines such as TNF-α and IL-12, regulate the ability of antigen presenting cells to T cells, as well as proliferation and differentiation of T lymphocytes and in the generation of regulatory T lymphocytes^5^ ^,^ ^6^. Studies have shown that butyrate may inhibit myeloperoxidase (MPO) activity attenuating the grade of inflammation in colonic mucosa, and regulates reactive oxygen species (ROS) generation in healthy patients and those with inflammatory bowel diseases (IBD)^7^ ^,^ ^8^. Also, butyrate induces the expression of the genes responsible by formation of mucins produced by goblet cells of the colonic glands^9^. The ability to decrease the inflammatory process of the colonic mucosa, the modulation of the ROS production and the increase in production of mucus confirm the importance of the regular supply of SCFAs to keep the colonic epithelial barrier integrity avoiding the development of colitis. Deficiency of SCFAs has been linked to the ethiopatogenesis of the IBD and diversion colitis (DC)^10^ ^-^ ^12^. 

 The colonic mucosal epithelial cells devoid of the regular supply of SCFAs are highly energy deprived, as indicated by decreased expression of enzymes involved in fatty acid metabolism in mitochondria^13^. Consequently, the cells of the colonic mucosa devoid from fecal stream suffer modifications of mitochondrial respiration which leads to an overproduction of ROS^14^ ^,^ ^15^. ROS are toxic to cells and their overproduction is capable of causing extensive oxidative damage to the synthesis of mucins that cover the colonic mucosa^11^ ^,^ ^16^. It was shown that increased oxidized molecules due to excessive oxidative stress in patients with IBD are correlated with severity of mucosal inflammation and damage to the mucus layer^16^ ^,^ ^17^. Studies adopting experimental models of DC also showed that oxidative stress, resulted from absence of SCFAs supply, reduce the population of goblet cells in the colonic mucosal glands and the content of different types of mucins aggravating the inflammation of the colonic mucosa^16^ ^,^ ^18^ ^,^ ^19^. Deficiencies in mucin layer that cover the colonic epithelium has been described in different intestinal diseases such IBD, irritable bowel syndrome, celiac diseases and DC^20^. Thus, the preservation of the mucus layer integrity is an essential factor in maintaining colonic mucosal homeostasis.

 Mesalazine (MEZ) has been used for treatment of patients with mild-moderate active ulcerative colitis (UC), radiation colitis and DC^21^ ^-^ ^23^. MEZ has multiple mechanisms of action with numerous anti-inflammatory properties including inhibition of cyclooxygenase, lipoxygenase, platelet-activating factor, interleukin 1, nuclear factor kappa beta, peroxisome proliferator-activated receptor-gamma and B-lymphocytes. MEZ due to its scavenger’s properties is a potent antioxidant reducing the overproduction of ROS by inflamed colonic mucosa^24^. Experimental study showed that use of enema containing MEZ can reduces the intensity of oxidative damage to epithelial barrier of colon segments without fecal stream, confirming the scavenging effects of MEZ against ROS^25^. Because oxidative stress is related to the damage of the mucus layer in experimental models of DC, it’s possible that use of enemas with MEZ neutralizing the overproduction of ROS, could reduce the inflammatory process and preserve the protection offered by mucins in colonic epithelium devoid of the fecal stream. However, to the best of our knowledge, no experimental study evaluate this possibility yet. Thus, the aims of the present study were to evaluate the effects of enemas with MEZ in the inflammatory process of the colonic mucosa and tissue content of different types of mucin in an experimental model of DC.

## Methods

 The experiments were performed in accordance with the principles outlined by Brazilian Federal Law nº 11.794 (10/08/2008) and were approved by the Ethics Committee in Animal Research of Universidade São Francisco (Nº 2211/2007).

### Animals and surgical techniques

 Twenty-four male Wistar rats (300-350g) were obtained from the ANILAB (ANILAB - Animais de Laboratório Criação e Comércio, Paulínea, São Paulo, Brazil), barrier facility and maintained on light/dark cycles of 12 hours, and fed a standard rodent chow diet. They were deprived of food, but not water, for 12h prior to the surgical procedure.

 Diversion of fecal stream was performed in all animals similar as previous described^25^. Briefly, animals were anesthetized by intramuscular administration of 0.1 ml/100 g 1:1 (v/v) solution of ketamine (50 mg/ml) and xylazine (20 mg/ml). The abdomen was shaved, and a 3-cm-long midline incision was made. The left colon was exteriorized and sectioned approximately 6 cm above the Peyer’s lymphoid patch located at the transition between the left colon and the rectum. Two circular fragments of skin, 3 mm in diameter and 3 cm apart, were made in the left side of the abdominal wall at the same vertical level. The proximal end of the colon was exteriorized through the cranial-cutaneous orifice, and, after splitting the abdominal wall muscles, the distal stoma was exteriorized through the caudal skin opening. The proximal end of the colon and the distal stoma were fixed to the skin with full-thickness 5-0 Prolene sutures (Ethicon Inc., Somerville, NJ, USA). Before fixation of the distal stoma to the skin, the distal colon was cleaned by infusing a physiologic solution until fecal contents were completely removed. The abdominal incision was closed in two stages: the muscle and aponeurosis were closed with 4-0 Vicryl (Ethicon Inc., Somerville, NJ, USA), and the skin was closed with 4-0 Prolene (Ethicon Inc., Somerville, NJ, USA). In this way, two colostomies were performed: the proximal colostomy, a terminal colostomy with intestinal transit; and the second colostomy, a distal stoma devoid of fecal stream. Rats were maintained in individual cages without special wound care given to the stomas or abdominal incisions.

### Experimental groups

**Figure 1:**
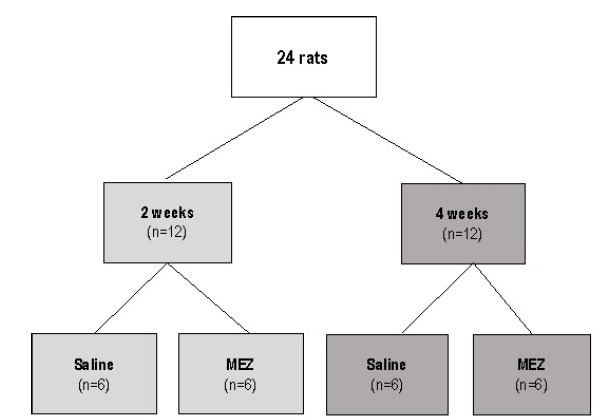
Algorithm of division of the experimental groups.

 Twenty-four animals were divided into two experimental groups with 12 animals each according the euthanasia had done after 2 or 4 weeks of treatment. These two experimental groups were divided into four subgroups with six animals each according to the intervention solution employed and time of intervention. In the first and second subgroups, six animals received daily rectal enemas containing 20 ml of saline (control subgroup) at 37ºC, and six received daily rectal enemas containing 20 ml of MEZ (Pentasa, Ferring laboratories, São Paulo, Brazil) at concentration of 100 mg/kg for 2 weeks. In the 12 remaining animals of the second group, six rats received daily rectal enemas with saline and six with MEZ at same concentration for 4 weeks. In order to standardize the speed and time of application, the enemas were administered in all animals with an infusion pump whose speed was standardized at 5/ml/min. Figure 1 show the algorithm of the division of the experimental groups.

### Sample collection

 Upon completion of the pre-determined irrigation period, the animals were anesthetized with the same technique used to diversion of the fecal stream, and the midline incision was opened again. In both groups, specimens were taken from the intra-abdominal part of the excluded colon (colon without fecal stream). The removed specimens of the colon without fecal stream, measuring approximately 6.0 cm each, was longitudinally opened through the anti-mesenteric border fixed in a piece of cork and referred to histological and histochemical analysis.

### Histological and histochemical analysis

 Fragments prepared for histological analysis were immersed in 10% neutral formalin buffer (Sigma-Aldrich, St. Louis, MO, USA) for 24 h, dehydrated by exposure to increasing ethanol concentrations and embedded in paraffin. Thereafter, sections of tissue were cut at 5 µm on a rotary microtome (Leica Biosystems, Nussloch, Germany), mounted on a glass slide, cleared, hydrated and stained with hematoxylin-eosin (HE) for evaluation of the presence of colitis. Slide analysis was performed with optical microscope (Eclipse DS-50, Nikon Inc., Osaka, Japan) with final magnification of x200. To establish the diagnosis of colitis, as well as the degree of inflammation, the histological slides were analyzed by two blinded observers. The diagnosis of colitis was made based on presence of five independent histological parameters: reduction of the crypt length, number of goblet cells, crypt abscess, intensity of neutrophil infiltration of the mucosa, and epithelial loss. These variables were stratified as crosses, according to the degree of each, as follows: 0: absent or no alterations; (+): when intensity was mild; (++): moderate and, (+++): intense. For all variables analyzed, the final value considered for each animal was the mean value after quantification of three distinct histological fields. The inflammatory score for each animal was obtained from the sum of the five variables analyzed.

 The tissue expression of the neutral and acid mucins was determined by histochemical technique of Periodic Acid Schiff (PAS) and Alcian-blue (AB), respectively, as previous report^16^. Neutral mucins stained magenta, while the acid mucins stained blue. To identify the subtypes of acid mucins (sulfomucins and sialomucins), the slides were stained using the high iron diamine-Alcian blue (HID-AB) histochemical technique, in accordance with the previous standardized methodology^17^. Through the HID-AB technique, sulfomucins stained brown and sialomucins stained blue. 

### Computer-assisted image processing

 The neutral and acid mucin content, as well the tissue content of sulfomucins and sialomucins, was quantified by means of computer-assisted image processing and was always performed in a focal field in which there were at least three complete and contiguous colonic glands, at a magnification of x200. A pathologist with experience of IBD, who was unaware of the origin of the material and the objectives of the study, evaluated the content of tissue expression of all types and subtypes of mucins. The images selected were captured on a video camera that had been coupled to an optical microscope. These images were processed and analyzed using the NIS-Elements 3.1 software (Nikon Inc., Osaka, Japan). By means of colored histograms in the red, green and blue (RGB) system, the software determined the color intensity and the number of pixels in each selected field, and the final data were transformed into percentage expression of mucins per field analyzed (%/field). The final value measured for each section was the mean of the values found from evaluating three different fields. The tissue content of sulfomucins and sialomucins was quantified in the same colonic glands.

### Statistical analysis

 The results obtained for the tissue contents of all types and subtypes of mucins were always described by the mean value with respective standard error. A significance level of 5% (p<0.05) was adopted for all tests. The Mann-Whitney test was used to compare the degree of inflammation, the tissue content of the all types of mucins of animals from the control (saline) and experimental (MEZ) groups and to compare colon segments submitted to intervention with saline or MEZ for two or four weeks. BioStat software (version 5.1) was used for the statistical analysis. Significant values when were compared colon segments without fecal stream irrigate with saline or MEZ, were marked with asterisk (*) when this level was less than 5% or two asterisks when this level was less than 1% . At the same way, the significant values found when the animals submitted to the intervention with saline or MEZ by two or four weeks were marked with one ticket (^•^) when this level was less than 5% or two tickets (^••^) when this level was less than 1%.

## Results

**Figure 2:**
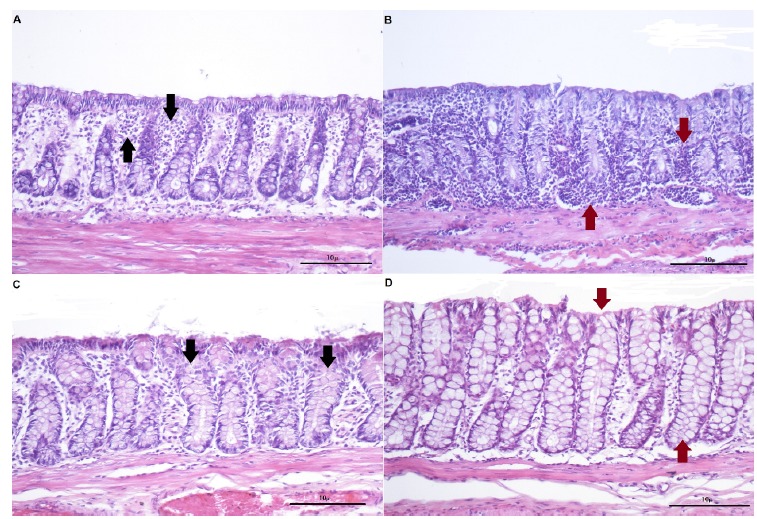
A: Colon epithelium devoid of fecal stream after intervention with saline by 2 weeks, It is possible verify the inflammatory infiltrate in colon mucosa (*black arrow*). B: Colon epithelium devoid of fecal stream after intervention with saline by 4 weeks. It is possible to observe the increase of the inflammatory cells in colon mucosa (*red arrow*). C: Colon epithelium without fecal stream in animals submitted to an intervention with MEZ by 2 weeks. We verify reduction of the inflammatory infiltrate and increase of the population of the goblet cells in colon glands (*black arrow*). D: Colon glands without fecal stream in animals submitted to an intervention with MEZ by 4 weeks. It is possible to verify an even greater increase in population of the goblet cells (*red arrow*) and reduction of the inflammatory infiltrate compared with those animals that receive MEZ by 2 weeks. (HE x200).

 In Figure 2A and B it is possible verify the colonic mucosa of the segments without fecal stream in animals submitted to intervention with saline for 2 or 4 weeks. After 2 weeks of intervention with saline t is possible to identify edema among colonic glands, inflammatory infiltrate in mucosa and, thickening of the muscularis mucosa layer. The inflammatory infiltrate it is more intense in colonic crypts submitted to intervention with saline after 4 weeks. Figure 2C and D show the colon segments without fecal stream in animals submitted to intervention with MEZ for 2 or 4 weeks. It is possible observer reduction of the edema and inflammatory infiltrate among colonic glands and increase in number of goblet cells. 

**Figure 3:**
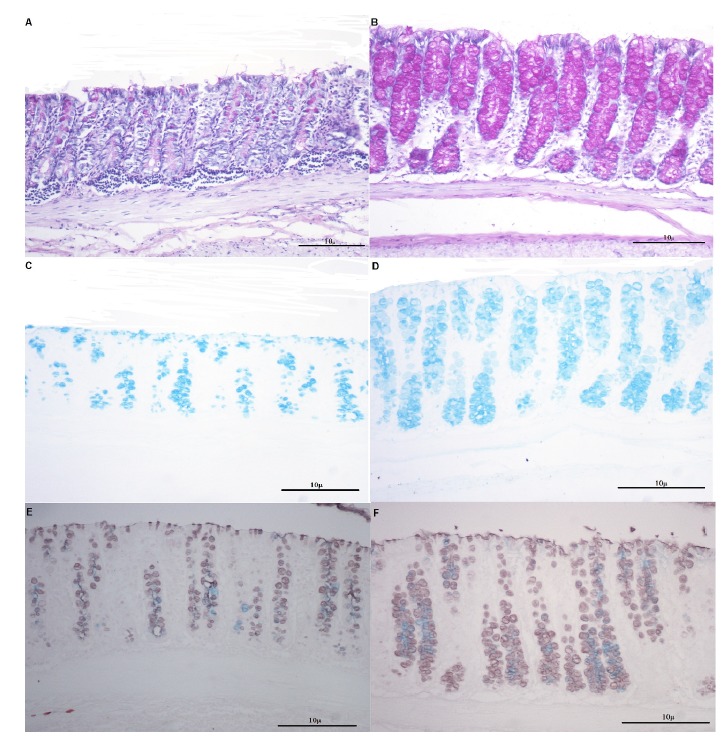
A: Reduction of the tissue expression of neutral mucins (staining in magenta) in glands of the colon epithelium devoid of fecal stream after intervention with saline by 4 weeks (PAS-x200). B: Increase in the tissue content of neutral mucins (staining in magenta) in goblet cells of the colon mucosa devoid of fecal stream submitted to an intervention with MEZ by 4 weeks (PAS-x200). C: Reduction of the tissue expression of acidic mucins (staining in blue) in goblet cells of the colon glands devoid of fecal stream after intervention with saline by 4 weeks (AB-x200). D: Increase in the content of the acidic mucins (staining in blue) in goblet cells of the colon glands devoid of fecal stream after intervention with MEZ by 4 weeks (AB-x200). E: Tissue expression of sulfomucins (staining in brown) and sialomucins (staining in blue) in goblet cells of the colon glands devoid of fecal stream after intervention with saline by 4 weeks (HIDAB-x200). F: Tissue expression of sulfomucins (staining in brown) and sialomucins (staining in blue) in glands of the colon epithelium devoid of fecal stream after intervention with MEZ by 4 weeks (HIDAB-x200). Increase in the sulfomucins and sialomucins content in the animals submitted to enemas with MEZ by 4 weeks in relation to those that received enemas with saline.

 In Figure 3 it is possible to verify the pattern tissue expression of neutral mucins, acid mucins, sulfomucins and sialomucins in colonic segments without fecal stream in animals submitted to intervention with saline or MEZ for four weeks. In Figure 3A and B it is possible to observe the pattern of tissue expression of neutral in glands of the colonic mucosa of animals submitted to intervention with saline or MEZ for 4 weeks. There are increases in tissue content of neutral mucins in colonic segments submitted to intervention with MEZ. In Figure 3C and D it is possible to see the pattern of tissue expression of acidic in glands of the colonic mucosa of animals submitted to intervention with saline or MEZ for 4 weeks. There are increases in tissue content of acidic mucins in colonic segments submitted to intervention with MEZ. In Figure 3E and F it is possible to observe the tissue content of sulfomucins and sialomucins in glands of the colonic mucosa of animals submitted to intervention with saline or MEZ for 4 weeks. There are increases in tissue content of both subtypes in animals submitted to intervention with MEZ.

 The mean values, with the respective standard error, for the inflammatory score, tissue content of neutral, acid mucins, sulfomucins and sialomucins, in colon segments without fecal stream, of the animals submitted to intervention with saline or MEZ, for two or four weeks are show in Table 1. It is possible to verify that animals submitted to intervention with saline presents high grade of inflammatory score compared those submitted to intervention with MEZ. The application of enemas with MEZ by 4 weeks showed more efficacies to reduce the grade of inflammation. Animals that received enemas with MEZ presents increase in the tissue content of neutral, acid and sufomucins when compare with those submitted to intervention with saline regardless of intervention time. However, in animals treated with MEZ, the content of sialomucins was only higher after 2 weeks of intervention than the animals that received intervention with saline.

**Table 1:** Mean value, with respective standard error, of the tissue content of neutral, acid mucins, sulfomucin and sialomucin in colon segments without fecal stream submitted to intervention with saline or MEZ for two or four weeks.

	2 weeks		4 weeks	
	Saline	MEZ	Saline	Mez
	M ± SE			
Inflammatory score	8.70±0.4**	5.31±0.3^••^	9.20±0.5**	3.30±0.3
Neutral mucin	6.67±0.34	15.25±0.98**	7.73±0.48	16.96±085**
Acid mucin	5.85±0.61	11.40±0.69**	9.22±0.46	19.84±2.35** ^••^
Sulfomucin	7.60±0.13	8.65±0.12**	6.58±0.21	15.55±0.85** ^••^
Sialomucin	0.04±0.006	0.27±0.05**	0.20±0.04^••^	0.33±0.01*

MEZ = Mesalazine. M = Mean. SE = Standard Error. * saline × MEZ (p<0.05); ** = saline × MEZ (p<0.01); ^••^ = 2 weeks × 4 weeks (p<0.01). Mann-Whitney test.

## Discussion

 The colonic mucosal barrier is made up of epithelial and immune cells which together form a barrier to harmful agents. The colonic epithelium is covered by a thick layer of mucus that serves as a first line of the colonic epithelium defense system^20^. The mucous layer represents one of the most important defense systems of the intestinal mucosa that prevents microorganism and noxious substances from reaching the surface of the epithelium. The protective effect provided by the mucous layer is related to the presence of a high molecular weight glycoprotein called mucins^16^. The mucins are produced and stored in granules of the goblet cells cytoplasm presents in colonic glands and are composed of two fractions, one glycid and other protein^16^ ^,^ ^17^ ^,^ ^19^. Regarding the protein fraction of the mucins molecule, several subtypes were described but the subtype MUC2 are the most often found in colonic epithelium. Others subtypes like MUC1, MUC4, MUC5A, MUC5B and MUC6 may also be expressed but in smaller quantities^9^ ^,^ ^20^. Regarding the glycid fraction, colonic mucins belong to two main histochemical groups: neutral and acid mucins^16^. Acid mucins are composed by two subtypes: sulfomucins and sialomucins^17^ ^,^ ^26^. Neutral mucins are expressed in greater quantities in the more cranial portions of the digestive tract and, going towards the colon, the predominant tissue expression changes to acid mucins^26^. 

 There are several evidences suggesting that SCFAs reinforces the colonic defense barrier mainly by stimulating the synthesis of mucins^3^ ^,^ ^7^. Studies have shown that butyrate increased the *MUC2* gene expression, stimulated mucin synthesis thereby increasing the thickness of the mucus layer that covers the colonic epithelium^8^ ^,^ ^9^. While the adequate supply of SCFAs to colonic mucosa increase the mucus production, studies has shown that in absence of a regular supply of SCFAs, like occur in colonic segments devoid of the fecal stream, there is a reduction in the tissue content and modification of the mucin expression pattern in glands of the colonic epithelium^8^ ^,^ ^16^ ^,^ ^18^. Thus, the colonic mucosa excluded from fecal stream becomes more vulnerable to the deleterious effects of overproduction of ROS and development of an inflammatory process^10^. 

 Deficiency of SCFAs colonic mucosa cells could potentially increase production of ROS, resulting in onset of mucosal inflammation ^6^ ^,^ ^10^ ^,^ ^15^ ^,^ ^17^ ^,^ ^18^. It is now well recognized that oxygen radicals such as superoxide, hydroxyl radical, hydrogen peroxide, and hypochlorous acid may be pathogenic and are produced in excess by colonic mucosa when devoid of fecal stream^10^ ^,^ ^15^. Increased formation of ROS and continuous exposure of colonic epithelium cells to these harmful radicals promote, damage of the mucus layer and intercellular adhesion molecules, peroxidation of cellular membrane lipids and oxidative DNA damage to epithelial cells, thereby triggering development of colitis and colorectal cancer^11^ ^,^ ^12^ ^,^ ^15^ ^,^ ^25^. The radical induction theory was proposed to explain the importance of the oxidative stress in the ethiopatogenesis of UC^15^.

 Experimental studies evaluated the effects of oxidative stress and substances with antioxidant properties on the tissue content of neutral an acidic mucins in the colonic mucosa with and without fecal stream^16^ ^,^ ^17^ ^,^ ^27^ ^-^ ^29^. The results showed that in colonic mucosa with fecal stream (provide of the regular supply of SCFAs) the tissue content of both types of mucins do not change along of colonic mucosa^16^. In colonic segments with fecal stream, the tissue content of neutral mucins is slightly higher than that of acidic mucins, especially in the proximal portions of the colon^16^ ^,^ ^26^. However, in the colonic segments devoid of fecal stream (without regular supply of SCFAs), there is a significant reduction in the content of both types of mucins, with a more pronounced reduction of the acidic mucins, mainly in the distal portions of the colon. The reduction of content of both types of mucins is related with increased levels of ROS and with the worst of mucosal inflammation and epithelial damage^10^ ^-^ ^13^ ^,^ ^16^ ^,^ ^17^. Although studies showed that in the colonic segments without fecal stream occurs a small increase in tissue content of both types of mucins with the time of colonic exclusion, these values are always smaller than content presents in the colon segments with fecal stream^16^ ^,^ ^17^. Thus, the regular supply of SCFAs protects the mucus layer that covers the colonic epithelium by two different mechanisms: stimulating the production of mucins by the goblet cells and reduce its damage by oxidative stress^13^ ^,^ ^19^. The influence of oxidative stress in reduction of the tissue content of neutral and acid mucins appear to be confirmed by experimental studies^27^ ^-^ ^29^. They showed that the application of enemas containing sucralfate or curcumin an antioxidant substance in colonic mucosa without fecal stream, in addition to reduce the oxidative stress and improve the inflammatory process, increased the tissue content of neutral and acidic mucins^27^ ^-^ ^29^. 

 The results of the present study seems to confirm the antioxidant action of MEZ in protect the colonic mucosa devoid of the fecal stream. Enemas with MEZ were able to reduce the mucosa inflammation and maintain the tissue content of neutral and acids mucins despite the lack of supply of SCFAs. Regardless of the time of intervention adopted, the tissue content of neutral and acidic mucins was always higher in animals treated with MEZ when compared with those treated with saline. There was an increase in the tissue content of acid mucins with the intervention time. Animals treated with MEZ also had an improvement in the inflammatory score when compared to those treated with saline. It is possible that these findings are related to the reduction of levels of oxidative damage. In a previous study, utilizing the same group of animals, we showed that the application of enemas with MEZ was able to reduce the levels of oxidative damage to the DNA of the isolate cells obtained from colonic segments devoid of fecal transit, even after *ex vivo* challenge with H_2_O_2_ ^25^. These results confirm the importance of the adequate supply of SCFAs to reduce the oxidative stress and consequently maintain the content of neutral and acidic mucins in colonic mucosa.

 Keli *et al.* ^26^ were the first to study the tissue content and the pattern of expression of the subtypes of acidic mucins (sulfomucins and sialomucins) in colonic segments diverted from fecal stream. The results showed that colonic segments without fecal stream presents reduction in the length of the colonic glands, increase of the inflammatory grade and significant modification of the pattern of distribution and tissue content of the two subtypes of acidic mucins. Using a subjective method of analysis the author has showed that sulfomucins, initially present in the upper two-thirds of the colic glands, with a weak intensity were found after 6 weeks of colonic diversion along of all colonic glands with a high intensity, and this was maintained at 17 weeks. Thus, in colonic mucosa devoid of the fecal stream sulfomucins is the predominant type of acidic mucins. Sialomucins were found only at 6 weeks in the inferior third of the gland with a high intensity and then at 17 weeks with a weak intensity all along the height of the gland^26^. However, the tissue content of both subtypes of acidic mucins was measure subjectively, therefore subject to variations in the measure between observers. Posteriorly, other’s authors using computer-assisted image analysis software to measure the content of both subtypes of acidic mucins showed that in the colonic mucosa without fecal transit there is a significant reduction in the tissue content of both subtypes when compared with mucosa with fecal stream^17^. Sialomucins showed a more significant reduction when compared with sulfomucins and practically disappear after 18 weeks of colonic diversion. As occur with neutral and total acid mucins, these same animals presents high inflammatory score and increase levels of oxidative stress in colonic segments without fecal stream, suggesting a relation among overproduction of ROS, mucosal damage and modification in tissue content of sulfomucins and sialomucins^10^ ^,^ ^17^. In order to evaluate the interference of oxidative stress on the tissue content of sulfomucins and sialomucins in colonic segments without fecal stream, experimental studies evaluated the effect of use of enemas containing antioxidant substances^25^ ^,^ ^27^ ^-^ ^30^. The results showed that sucralfate, n-acetylcysteine and, oil extract of curcimun, substances with antioxidant properties, in addition to reducing the degree of tissue inflammation, and the levels of lipids peroxidation, increase the content of both acidic mucin subtypes in the colonic mucosa without supply of SCFAs.

 The results of this study seems to confirm the action of MEZ in maintain the tissue content of sulfomucins and sialomucins in colonic mucosa devoid of the fecal stream. Regardless of the time of intervention adopted, the tissue content of sulfomucins and sialomucins mucins was always higher in animals treated with MEZ when compared with those treated with saline. There was an increase in the tissue content only of sulfomucins with the intervention time. Although the sialomucins are the subtype of acid mucin that presents the greatest reduction of its contents in colonic mucosa excluded from fecal stream, the tissue content of sialomucins was always higher in animals treated with MEZ when compared with those treated with saline. These same results were found by other authors who used enemas with antioxidant substances in the mucosa devoid of fecal stream^29^ ^,^ ^30^. Taken together, all these results suggest that the tissue content of both acidic mucin subtypes increases with the regular supply of SCFAs and with the reduction of tissue oxidative stress. 

 These results reinforce the possibility that oxidative stress caused by overproduction of ROS by epithelial cells devoid of fecal stream may be related to the reduction of the mucins contents. The data confirm the benefits of MEZ in the treatment of DC, as demonstrated by its therapeutic effects at increasing the production of the all types and subtypes of mucins studied. Previous experimental studies showed that the use of enemas containing MEZ could reduce levels of oxidative damage in cells of chronically inflamed colonic mucosa, and may be this one of the most important mechanism related to reduction of the mucins content^25^. Because oxidative damage caused by ROS is one of the mechanisms related to rupture of the epithelial barrier in patients with IBD and DC, it is possible that antioxidant activity preserving the mucins contents is an explanation for the protective effects of MEZ in the treatment of these diseases^7^ ^,^ ^11^ ^,^ ^12^ ^,^ ^17^ ^,^ ^23^ ^,^ ^25^ ^,^ ^26^. Although these data demonstrate the efficacy of MEZ in preserve the tissue content of the mucins in the colonic epithelium devoid from fecal stream in experimental model of DC, further studies in patients with DC should be performed to confirm whether these effects are replicated in humans.

## Conclusion

 Daily enemas with MEZ can improve the inflammatory process and increase the tissue content of mucins in colonic mucosa devoid of the fecal stream in an experimental model of diversion colitis.
